# The utility of brain CT scan modality in the management of dizziness at the emergency department: A retrospective single-center study

**DOI:** 10.1016/j.amsu.2021.102220

**Published:** 2021-03-13

**Authors:** Khaled Z. Alawneh, Liqaa A. Raffee, Ahmad A. Oqlat, Ammar A. Oglat, Majdi Al Qawasmeh, Musaab K. Ali, Anas M. Okour, Abdel-Hameed Al-Mistarehi

**Affiliations:** a Department of Diagnostic Radiology and Nuclear Medicine, Faculty of Medicine, Jordan University of Science and Technology, Irbid, Jordan; b Department of Accident and Emergency Medicine, Faculty of Medicine, Jordan University of Science and Technology, Irbid, Jordan; c Emergency Medicine Specialist, Department of Accident and Emergency Medicine, Faculty of Medicine, Jordan University of Science and Technology, Irbid, Jordan; d Department of Medical Imaging, Faculty of Applied Medical Sciences, The Hashemite University, Zarqa, Jordan; e Division of Neurology, Department of Neurosciences, Faculty of Medicine, Jordan University of Science and Technology, Irbid, Jordan; f Emergency Medicine Specialist, Department of Emergency Medicine/Emergency Medicine Fellow, King Abdullah University Hospital, Jordan /Faculty of Medicine and Health Sciences, Omdurman Islamic University, Sudan; g Emergency Medicine Resident, Department of Accident and Emergency Medicine, Faculty of Medicine, Jordan University of Science and Technology, Irbid, Jordan; h Department of Public Health and Family Medicine, Faculty of Medicine, Jordan University of Science and Technology, Irbid, Jordan

**Keywords:** Dizziness, Vertigo, CT scan, MRI, Effectiveness, Emergency

## Abstract

**Objectives:**

This study examines the usefulness of computed tomography (CT) scans in evaluating patients with dizziness in the emergency department (ED).

**Methods:**

Medical records of patients presented with complaints of dizziness or vertigo to the ED of a tertiary university hospital and underwent head CT scans from July 2015 to June 2018 were reviewed. The patients’ demographic information, presenting symptoms, and final head CT scan and Magnetic resonance imaging (MRI) results were collected. Stepwise logistic regressions were used to analyze data.

**Results:**

A total of 326 dizzy patients were included in this study. The majority of the patients (83.1%) were older than 44 years. Acute vertigo pattern of dizziness was detected among 50.6% of the patients and was more common among females than males (*p* < 0.001). Of these 326 patients who underwent head CT scans, 49 (15%) had abnormal findings with acute ischemic stroke was the most common one. A total of 191 patients underwent follow-up studies. MRI accounted for 70% of the follow-up studies. Of the 134 patients who received MRI of the brain, 36 (27%) had abnormal findings. A significant correlation of RBCs level, presence of other symptoms, and frequency of episodes with the presence of vertigo (*p* < 0.001) was found.

**Conclusion:**

The study's findings indicate low effectiveness of head CT scan compared to MRI for dizziness management. Future studies are suggested to provide more insights into the cost-effectiveness and utility of head CT scans and MRI in providing valuable findings.

## Highlights

• The majority of the dizzy patients presented to the emergency were older than 44 years.
• Acute vertigo was detected among 50.62% of the patients and more common among elderly females.
• Lower effectiveness of CT scan compared to MRI in the diagnosing and management of dizziness.
• Significant correlation of RBCs level, presence of other symptoms, and frequency of episodes with the presence of vertigo.

## 1 Introduction

Dizziness is a vague and unspecified term used by patients to describe the sensation or illusion of movement, disequilibrium, or unsteadiness [1]. It is explained under three different categories included: Syncope, Vertigo, and Disequilibrium. Patients with vertigo may sense rotation and forward-backward movement during their normal working [2]. In contrast, disequilibrium is the patient's inability to balance the torso and is not significantly accumulated by adjusting movement [3]. For syncope, history of death in any family member aged <30 years, tonic-clonic movements, and loss of consciousness distinguish it from disequilibrium and vertigo [2]. The occurrence of psychiatric problems and peripheral lesions was categorized as more than 70% of dizziness causes [4].

Previous studies have found that laboratory tests such as evaluating lipid profile and brain computerized tomographic (CT) scan were unjustifiable [5,6]. Saccomano et al. claimed that several dizziness cases might be distinguished through a comprehensive history and physical examination of patients [2]. In the US, dizziness is one of the most common complaints in the emergency department (ED) and is responsible for 2.6 million and 3.3% of all ED visits [7]. Uncommonly, 40% of Americans look for medical awareness for dizziness at a certain age during their lifetime [8]. Therefore, the estimation and evaluation of dizziness highly contribute to the health care cost. The overall cost of care for treating patients with balance disorders is over $1 million, and the patient-care cost for balance disorder-related falls is more than $8 billion per year [[9], [10], [11]].

Dizziness is viewed as an enigma, where most of the patients face difficulty articulating the etiologies and other numerous symptoms. Other essential factors that are considered problems associated with the disease are physicians' limited knowledge, time-related limitations, and the prevailing pressure upon them regarding dizziness as a life-threatening disorder. In ED, this occurrence resides in rapidly examined cases that need emergency care (intracranial bleeding, stroke, and myocardial infarction), identification of the operative pathology that develops the symptoms, and the site of lesion (tumor), and timely treatment through a concerned doctor is often helpful.

To fit within the given limitations, these patients are provided with a detailed evaluation, including physical examination, routine-based imaging (MRI, CT scan of the head/brain), and laboratory testing. Head CT scan is frequently used as a first-line imaging modality to exclude posterior fossa hemorrhage or a large mass as a cause for vertigo, although there is a substantial variation between EDs in their imaging practices [12]. Previous studies indicated a low diagnostic yield for head CT scan in dizzy patients [13,14]. Besides, a low CT scan sensitivity in diagnosing ischemic strokes which is a common cause of dizziness [15]. This study aims to examine the usefulness of head CT scans in evaluating patients with complaints of dizziness or vertigo who were presented in the ED of a tertiary hospital.

## 2 Materials and methods

### 2.1 Study design and setting

This study follows a retrospective design. Any adult patient presented to the ED of King Abdullah University Hospital (KAUH), Irbid, Jordan, during the period from July 2015 to June 2018, and received a head CT scan to evaluate a complaint of dizziness or vertigo was included in the study. As not to miss any data on dizziness and as the terms of ‘vertigo’ or ‘dizziness’ were often used interchangeably; therefore, the electronic query of the head CT scan patients' database for the terms of ‘vertigo’ or ‘dizziness’ were used to identify the included patients. Exclusion criteria were children (less than 18 years old), patients who had a history of recent neurosurgery, a predisposing neurological disorder, or migraine, and dizzy patients who did not receive a head CT scan within three months of their presentation to the ED. Also, patients were excluded if the provider notes for the accompanying encounter made no mention of the sensation of spinning or translational motion (in the case of vertigo) or descriptors equivalent to lightheadedness, disequilibrium, or presyncope (in the case of dizziness).

Using the standard methodology, patients’ charts were reviewed, particularly those who had head CT scans to indicate vertigo/dizziness at the selected institution. The study was based on patients with dizziness/vertigo, with or without physical examinations, along with those referred for head CT scan when presented in the ED department either within three months of the ED visit or during the index hospitalization. Medical records of patients were also reviewed. The initial outcome of the vertigo was finalized through the documentation presented by the neurology and acute stroke.

### 2.2 Clinical data collection

Data were collected by reviewing the electronic medical records, radiological reports, and discharge summaries. The collected information included details regarding patients' sex, age, race, illicit drugs and tobacco use, and medical history of diseases. The history of smoking and diseases such as cancer, dysrhythmia, hypertension, benign paroxysmal positional vertigo, and ischemic heart disease (IHD) were also included. Also, patients’ history of complaints regarding the present illness such as; dizziness, near-syncope, lightheadedness, and vertigo were evaluated. Along with it, findings of the physical examination were collected. Neurological findings documented by the neurology consult team either during the examination or by the indication for the consult were reviewed. Follow-up data within three months after the ED visit were also reviewed and collected, as some of them were recommended for MRI in the follow-up visit to the neurology department.

### 2.3 Imaging data collection and analysis

The neuroradiologists' clinical reports were used to collect the initial head CT scan results and any follow-up imaging such as MRI, CT scan, MRA, etc. Follow-up imaging was coded by findings and modality based on the acuity and the impact on diagnosis associated with the initial head CT scan. The head CT scan's positive findings were then confirmed through chart reviews undertaken by two independent investigators.

### 2.4 Data analysis

The neurology diagnosis at the time of discharge was considered a conclusive diagnosis. However, the overall data were recorded in an electronic database (Access 2003, Microsoft Corporation, Redmond, WA), then transferred for analysis in the Statistical Package of Social Sciences (SPSS) version 23.0. Frequencies and percentages of the variables were calculated. A Pearson chi-square test was conducted to find an association between dizziness and associated factors with respect to gender. Furthermore, logistic regression analysis was done to find the significance of the variables. Here, RBCs level, presence of other symptoms, and frequency of episodes were the independent factors (chosen predictive factors), and dizziness pattern was the dependent variable.

### 2.5 Ethics statement and work protocol

Ethical approval from the Institutional Review Board (IRB) at Jordan University of Science and Technology was obtained before commencing the study. Ethical approval from the radiology department of KAUH was further obtained. The study ensured to anonymize the information collected from different patients throughout the study period. This study was conducted following the 1975 Helsinki declaration, as revised in 2008 and its later amendments or comparable ethical standards [16]. This work has been reported in line with the STROCSS 2019 criteria (Strengthening the Reporting of cohort studies in surgery) [17]. The protocol had been registered at the Research Registry website with the registration unique identifying number of researchregistry6540 [18].

## 3 Results

A total of 326 dizzy patients were included in this study. Table 1 provides demographic characteristics of patients. Findings indicated that 52.5% of patients were females, and 47.5% were males. The majority of the patients (83.1%) were older than 44 years. Regarding comorbidities, 40.2% of patients had diabetes mellitus comorbidity, followed by a history of stroke (33.7%) and hypertension (20.6%). Other highlighted diseases were asthma (1.2%), depression (1.2%), dyslipidemia (1.5%) and IHD (1.5%). 12.9% of patients were using beta-blockers, while 12.3% of patients were using diuretics. Findings to the dizziness pattern indicated that 50.62% of patients sensed vertigo, while 40.18% of the patients sensed non-specific dizziness. 21.5% of patients indicated multiple episodes for the frequency of dizziness pattern. In comparison, only 17.5% were identified with single episodes.

**Table 1:** Demographic characteristics of patients.

Variable		Frequency	Percent
Gender			
	Male	155	47.5
	Female	171	52.5
Age Group			
	18–30 years	27	8.3
	31–43 years	28	8.6
	44–56 years	89	27.3
	57–69 years	78	23.9
	70–82 years	89	27.3
	>83 years	15	4.6
Comorbidities			
	Asthma	4	1.2
	Depression	4	1.2
	DM	131	40.2
	Dyslipidemia	5	1.5
	HTN	67	20.6
	IHD	5	1.5
	Hx of stroke	110	33.7
Chronic Medications			
	ACE Inhibitors	21	6.4
	ARBs	14	4.3
	Aspirin	25	7.7
	Atorvastatin	8	2.5
	Beta-blockers	42	12.9
	Calcium channel blockers	9	2.8
	Diuretics	40	12.3
	Oral diabetic	10	3.1
Smoking Status			
	Current smoker	24	7.4
	Ex-smoker	20	6.1
	Non-smoker	282	86.5
Dizziness Pattern/Description			
	Vertigo	165	50.62
	Lightheadedness	18	5.52
	Weakness	6	1.84
	Headache	6	1.84
	Non-specific Dizziness	131	40.18
Frequency of Episodes			
	Multiple episodes	70	21.5
	Single episodes	57	17.5
	NA	199	61.0

Abbreviations: DM, diabetes mellitus; HTN, hypertension; IHD, ischemic Heart Disease; ACE inhibitors, angiotensin-converting enzyme inhibitors; ARBs, angiotensin receptor blockers; NA, Not known.

Table 2 shows the clinical characteristics of the included patients. 90.8% of the patients showed normal RBCs level; 79.8% showed normal Hb level; 83.4% had normal creatinine level, and urea level was normal among 85.9% of patients. Also, findings related to normal Na, K, and Ca levels were observed among 95.4%, 96.3%, and 94.2% of patients, respectively.

**Table 2:** Laboratory findings of patients.

Laboratory test		Frequency	Percentage
RBCs level			
	High	5	1.5
	Normal	296	90.8
	Low	25	7.7
Hb level			
	Normal	260	79.8
	Low	66	20.2
Creatinine Level			
	High	37	11.3
	Normal	272	83.4
	Low	17	5.2
Urea Level			
	High	27	8.3
	Normal	280	85.9
	Low	19	5.8
Sodium (Na) level			
	Normal	311	95.4
	Low	15	4.6
Potassium (K) level			
	High	3	.9
	Normal	314	96.3
	Low	9	2.8
Calcium (Ca) level			
	High	7	2.1
	Normal	307	94.2
	Low	12	3.7
CK level			
	High	13	4.0
	Normal	123	37.7
	NA	190	58.3
CK-MB level			
	High	14	4.3
	Normal	118	36.2
	NA	194	59.5
Troponin level			
	High	11	3.4
	Normal	83	25.5
	Low	1	0.3
	NA	231	70.9
PT (prothrombin time) level			
	High	11	3.4
	Normal	85	26.1
	Low	1	0.3
	NA	229	70.2
INR level			
	High	7	2.1
	Normal	90	27.6
	Low	1	0.3
	NA	228	69.9
PTT (partial thromboplastin time) level			
	High	2	0.6
	Normal	68	20.9
	Low	3	0.9
	NA	253	77.6

Abbreviations: RBCs, red blood corpuscles; Hb, hemoglobin; CK, creatine kinase; CK-MB, creatine kinase-myoglobin binding; INR, international normalized ratio.

Table 3 provides results for the effects of gender. Vertigo pattern of dizziness was more common among females than males (*p* < 0.001). Diabetic Mellitus was the commonest comorbidity reported among male and female patients with the given values of (39.4%) and (40.9%) respectively. Results concerning dizziness episodes indicated that multiple dizziness episodes were reported among 21.3% male and 21.6% female participants, whereas single dizziness episode was higher in males (19.4%) than females (15.8%).

**Table 3:** Gender effects.

		Gender		*p-value*
		Male	Female	
Vertigo	No	98	63	*<0.001*
		63.2%	36.8%	
	Yes	57	108	
		36.8%	63.2%	
Comorbidities	Asthma	3	1	*0.127*
		1.9%	0.6%	
	Depression	1	3	
		0.6%	1.8%	
	DM	61	70	
		39.4%	40.9%	
	Dyslipidemia	5	0	
		3.2%	0.0%	
	HTN	31	36	
		20.0%	21.1%	
	IHD	4	1	
		2.6%	0.6%	
	Stroke	50	60	
		32.3%	35.1%	
Frequency of dizziness	Multiple episodes	33	37	*0.693*
		21.3%	21.6%	
	Single episodes	30	27	
		19.4%	15.8%	
	NA	92	107	
		59.4%	62.6%	

Table 4 shows neuroimaging findings of dizzy patients. Of these 326 patients who underwent head CT scans, more than two-thirds of the patients (85%) were reported with normal findings, while 49 (15%) had abnormal findings with acute ischemic stroke was the most common one (7%). A total of 191 patients underwent follow-up studies out of 326 participants. About one-third of the patients (41%) were referred to MRI investigations, which accounted for 70% of the follow-up studies. Of the 134 patients who received MRI of the brain, 36 (27%) had abnormal findings.

**Table 4:** Neuroimaging of patients.

		Frequency	Percentage
**Findings in non-enhanced brain CT-scan (total n = 326)**			
	Acute ischemic stroke	22	6.74
	Space-occupying mass	5	1.53
	Hemorrhage	6	1.84
	Previous infarction	16	4.90
	No findings/Normal	277	84.97
**MRI (total n = 134)**			
	Positive findings	36	26.87
	No findings/Normal	98	73.13
**Three-months follow-up studies**			
	Yes	191	58.59
	No	135	41.41
**Three-months Follow-up brain neuroimaging studies (after the initial CT scan)**			
	Another CT scan	23	7.05
	CTA	18	5.52
	MRA	16	4.90
	MRI	134	41.11
	No follow-up imaging	135	41.42

Abbreviations: CT scan, computed tomography scan; MRI, magnetic resonance imaging; CTA, computed tomography angiography; MRA, Magnetic resonance angiography.

Table 5 displays stepwise regression analysis for the presence of vertigo among patients. The findings showed a significant correlation of RBCs level, presence of other symptoms, and frequency of episodes with vertigo (*p* < 0.001).

**Table 5:** Stepwise regression analysis for the presence of vertigo among patients.

Model		Unstandardized Coefficients		Standardized Coefficients	t	*p-value*
		B	Std. Error	Beta		
1	(Constant)	0.196	0.092		2.130	*0.034*
	Symptoms (other than dizziness)	0.046	0.013	0.193	3.537	*<0.001*
2	(Constant)	0.569	0.136		4.186	*<0.001*
	Symptoms (other than dizziness)	0.047	0.013	0.197	3.682	*<0.001*
	RBCs level	−0.149	0.041	−0.196	−3.670	*<0.001*
3	(Constant)	0.715	0.151		4.747	*<0.001*
	Symptoms (other than dizziness)	0.046	0.013	0.195	3.659	*<0.001*
	RBCs level	−0.138	0.041	−0.181	−3.371	*0.001*
	Frequency	−0.072	0.033	−0.118	−2.194	*0.029*

a. Dependent Variable: vertigo.

## 4 Discussion

This study provided a detailed analysis of the effectiveness of the brain CT scan modality in dizziness management. Our findings indicate the low effectiveness of head CT scans in dizziness management among ED patients as MRI reported better diagnosis for dizziness evaluation. Also, vertigo pattern of dizziness was more common in elderly females.

Staibano et al. observed a significant proportion of females for dizziness [19]. Women are believed to be more sensitive to dizziness and imbalance and feel a greater influence on their daily activities. A study conducted by Neuhauser et al. has also confirmed this finding that females are reported with the lifetime occurrence of either severe or moderate dizziness [20]. Furthermore, such females experienced an increased degree of psychiatric comorbidity and disability compared to males [21].

The majority of patients reported in this study were older than 44 years. In the study of Staibano et al. most of the patients were 51–60 years old, which shows a similar pattern in terms of demographic characteristics [19]. Previous studies have also explained a pattern of demographic characteristics, which indicated most of the patients were elderly and a predominance of female patients [[22], [23], [24], [25]].

The findings of this study reported multiple episodes of dizziness among both male and female patients. Kerber et al. outlined that patients usually have multiple dizziness episodes, followed by imbalance, nausea, and vomiting [15]. These episodes were associated with either hearing loss, severe ear fullness, or loud roaring tinnitus and generally lasted for hours [15]. The lack of evidence on the prevalence of the first episode of dizziness has been observed, which potentially led to underestimating dizziness as a symptom. This finding recommends that improvements should be made to diagnose and manage dizzy patients in an emergency setting.

Our findings have shown a little diagnostic value obtained from head CT scans. Previously, similar findings reported that a head CT scan might not be cost-effective and yielded a low diagnostic value for significant pathology [13,26,27]. In this retrospective study, the majority of the patients showed better diagnostic value through MRI. It must be underlined that dizziness is a non-specific and common symptom and might be motivated by several disorders such as vestibular and multisensory, neurological, and cardiovascular dysfunctions.

Diabetic Mellitus and stroke are amongst the comorbidities in 40.2% and 33.7% of patients with dizziness, respectively. In contrast, depression was diagnosed in less than 5% of patients who presented with dizziness. Staibano et al. has confirmed that patients' condition of dizziness was associated with comorbid psychiatric disorders such as depression and anxiety in emergency settings [19]. Previous studies suggested that psychological intervention could significantly improve dizziness-related symptoms, disability, and functional impairment among dizzy patients [28,29]. Our cohort's low percentage of depression could be attributed to the little attention to psychiatric disorders and underdiagnosis of depression in ED.

Karatas's review indicated that the most common causes of vertigo were cerebrovascular disorders, migraine, multiple sclerosis, brain tumors, neurodegenerative disorders, some drugs, and psychiatric disorders with a little attention to cardiac causes [30]. Our findings indicated that the extent of IHD was low, which contradicts the previous studies' findings [[31], [32], [33]]. In these previous studies, a high percentage of patients had syncope or falls in addition to dizziness or the dizziness described as lightheadedness, while the percentage of such symptoms among our cohort was 5.5% which explains the low extent of IHD. Several studies suggest healthcare workers do not consider cardiovascular causes when a patient reports true vertigo (spinning/motion) as opposed to presyncope (lightheadedness) [31,[34], [35], [36]]. Two studies, one in a primary care clinic and another in an emergency department, did not find any diagnostic electrocardiographic changes in dizzy patients [37,38].

Furthermore, this study found that beta-blockers were commonly used by patients with dizziness, followed by diuretics. One of the known side effects of beta-blockers is dizziness, as they slow down the heart rate and trigger symptoms associated with low blood pressure (hypotension) by blocking the effects of epinephrine. It is also known that antihypertensive drugs may also cause vertigo or dizziness as an adverse effect, more precisely calcium channel blockers, angiotensin receptor blockers, and thiazide diuretics [[39], [40], [41], [42]].

Contributions of the study are significant for both patients and medical experts. As for patients, the study provides important insights regarding head CT scan usefulness during such health conditions. For clinical experts, this study serves as a guideline to improve the use of techniques for patients' quality healthcare. The study's findings are further useful in developing policies regarding treatment modalities, specifically in Jordanian hospitals for improved healthcare services.

Several limitations in this study are noteworthy. Recall bias may have occurred, which led to an underestimation of remote episodes of dizziness. Further, this retrospective study is limited since the collected data is small due to the lack of correct medical records, including the probabilities of patients' unattended historical information. Finally, this study did not focus on information related to the different types of dizziness.

## 5 Conclusion

The identification of vertigo in most patients presented to the emergency department was common among women over 45 years of age. The findings have shown little diagnostic value obtained from head CT scans among dizzy patients. On the contrary, MRI was found to be a more accurate imaging modality than CT scans to assess dizziness. It must be underlined that dizziness is a non-specific and common symptom and might be motivated by several disorders such as vestibular and multisensory, neurological, and cardiovascular dysfunctions. The findings have suggested development in the management and evaluation of dizziness among patients presented in the emergency department. Moreover, the findings offer effective and unique approaches for diagnosing and managing patients with dizziness of several etiologies.

Future researchers are suggested to conduct a study by providing more insights for the cost-effectiveness and utility of head CT scan in providing the valuable findings. They are further suggested to provide the comparative analysis of patients’ clinical characteristics presented with dizziness and those with acute head CT scan findings. This may help in the identification of patients and their associated needs of imaging. Finally, a prospective study is suggested to identify the difference in imaging frequency taking place by imaging algorithm intervention before and after the test.

## 6 Footnote page

### 6.1 Compliance with ethical standards

All procedures performed in this study involving human participants were reviewed and ethically approved by the Institutional Review Board (IRB) and research and ethics committee at Jordan University of Science and Technology. This study was conducted following the 1975 Helsinki declaration, as revised in 2008 and its later amendments or comparable ethical standards. This work has been reporting based on STROCSS 2019 guidelines (Strengthening the Reporting of cohort studies in surgery).

### 6.2 Informed consent

Informed consent was waived due to the retrospective nature of the study.

Research registration unique identifying number (UIN):

Name of the registry: Research Registry.

Unique Identifying number or registration ID: researchregistry6540.

Hyperlink to the specific registration:

https://www.researchregistry.com/register-now#user-researchregistry/registerresearchdetails/601f05f02a5ecb001bc012ad/

## Availability of data and materials

The datasets generated and analyzed during the current study are available with the corresponding author.

## Funding

No Funding was received for this study through any source.

## Ethical approval

Institutional approval was obtained from the Institutional Review Board (IRB) at Jordan University of Science and Technology.

## Sources of funding for your research

No funding.

## Author contribution

All authors contributed significantly and in agreement with the content of the article. All authors were involved in project design, data collection, analysis, statistical analysis, data interpretation and writing the manuscript. All authors presented substantial contributions to the article and participated of correction and final approval of the version to be submitted.

## Consent

Written informed consent was waived due to the retrospective nature of the study.

## Registration of research studies

UIN: researchregistry6540

https://www.researchregistry.com/register-now#user-researchregistry/registerresearchdetails/601f05f02a5ecb001bc012ad/

## Guarantor

Khaled Z. Alawneh.

Associate professor.

Department of Diagnostic Radiology and Nuclear Medicine, Faculty of Medicine, Jordan University of Science and Technology, Irbid, Jordan. Email: kzalawneh0@just.edu.jo.

## Provenance and peer review

Not commissioned, externally peer-reviewed.

## Declaration of competing interest

The authors declare that they have no competing interests.
